# Tracking the Molecular Scenarios for Tumorigenic Remodeling of Extracellular Matrix Based on Gene Expression Profiling in Equine Skin Neoplasia Models

**DOI:** 10.3390/ijms23126506

**Published:** 2022-06-10

**Authors:** Przemysław Podstawski, Katarzyna Ropka-Molik, Ewelina Semik-Gurgul, Marcin Samiec, Maria Skrzyszowska, Zenon Podstawski, Tomasz Szmatoła, Maciej Witkowski, Klaudia Pawlina-Tyszko

**Affiliations:** 1 Department of Animal Molecular Biology, National Research Institute of Animal Production, Krakowska 1 Street, Balice, 32-083 Kraków, Poland; ewelina.semik@iz.edu.pl (E.S.-G.); tomasz.szmatola@iz.edu.pl (T.S.); klaudia.pawlina@iz.edu.pl (K.P.-T.); 2 Department of Animal Reproduction, Anatomy and Genomics, University of Agriculture in Kraków, Mickiewicza 24/28, 30-059 Kraków, Poland; zenon.podstawski@urk.edu.pl; 3 Department of Reproductive Biotechnology and Cryoconservation, National Research Institute of Animal Production, Krakowska 1 Street, Balice, 32-083 Kraków, Poland; marcin.samiec@iz.edu.pl (M.S.); maria.skrzyszowska@iz.edu.pl (M.S.); 4 Center for Experimental and Innovative Medicine, University of Agriculture in Krakow, Rędzina 1c Street, 30-248 Kraków, Poland; 5 Institute of Veterinary Medicine, University Centre of Veterinary Medicine JU-AU, Mickiewicza 24/28, 30-059 Kraków, Poland; mawitkow@gmail.com; 6 Horse Clinic Służewiec, Puławska 266 Street, 02-684 Warsaw, Poland

**Keywords:** domestic horse, dermal tissue, molecular pathway, ECM remodeling, cell adhesion, sarcoid, procancerous tumorigenesis, RNA-seq

## Abstract

An important component of tissues is the extracellular matrix (ECM), which not only forms a tissue scaffold, but also provides the environment for numerous biochemical reactions. Its composition is strictly regulated, and any irregularities can result in the development of many diseases, including cancer. Sarcoid is the most common skin cancer in equids. Its formation results from the presence of the genetic material of the bovine papillomavirus (BPV). In addition, it is assumed that sarcoid-dependent oncogenic transformation arises from a disturbed wound healing process, which may be due to the incorrect functioning of the ECM. Moreover, sarcoid is characterized by a failure to metastasize. Therefore, in this study we decided to investigate the differences in the expression profiles of genes related not only to ECM remodeling, but also to the cell adhesion pathway, in order to estimate the influence of disturbances within the ECM on the sarcoid formation process. Furthermore, we conducted comparative research not only between equine sarcoid tissue bioptates and healthy skin-derived explants, but also between dermal fibroblast cell lines transfected and non-transfected with a construct encoding the E4 protein of the BP virus, in order to determine its effect on ECM disorders. The obtained results strongly support the hypothesis that ECM-related genes are correlated with sarcoid formation. The deregulated expression of selected genes was shown in both equine sarcoid tissue bioptates and adult cutaneous fibroblast cell (ACFC) lines neoplastically transformed by nucleofection with gene constructs encoding BPV1-E1^E4 protein. The identified genes (*CD99*, *ITGB1*, *JAM3* and *CADM1*) were up- or down-regulated, which pinpointed the phenotypic differences from the backgrounds noticed for adequate expression profiles in other cancerous or noncancerous tumors as reported in the available literature data. Unravelling the molecular pathways of ECM remodeling and cell adhesion in the in vivo and ex vivo models of epidermal/dermal sarcoid-related cancerogenesis might provide powerful tools for further investigations of genetic and epigenetic biomarkers for both silencing and re-initiating the processes of sarcoid-dependent neoplasia. Recognizing those biomarkers might insightfully explain the relatively high capacity of sarcoid-descended cancerous cell derivatives to epigenomically reprogram their nonmalignant neoplastic status in domestic horse cloned embryos produced by somatic cell nuclear transfer (SCNT).

## 1. Introduction

The extracellular matrix (ECM) is an important component of every tissue, which apart from forming its scaffold, also provides an appropriate environment for a number of biochemical processes, thus enabling the maintenance of homeostasis of the organism [1,2]. Each tissue has its own ECM composition, but its basic components are water, proteins and polysaccharides [1]. These components enable the control of their behavior through their continuous interaction with cells in several processes, such as migration, adhesion, proliferation, differentiation, and survival [2,3]. In addition, the components of the ECM are tightly organized and constantly change as a result of biochemical processes within the ECM that must be carefully controlled. Any uncontrolled changes in the composition of these components may lead to disturbances in the functioning of the whole organism, thus leading to the development of disease [2,3]. In humans, these changes in the ECM are associated with many diseases, such as osteogenesis imperfecta, Marfan syndrome, coronary heart disease, hypertension, and asthma, as well as diseases of other systems (liver cirrhosis, inflammatory bowel diseases, chronic kidney diseases) [3]. Moreover, pathological changes in the composition of the ECM are considered to be one of the most important factors leading to cancer.

In equines, the most common skin tumor is the sarcoid. This neoplasia is characterized by a lack of metastatic capacity, although it may disturb the well-being of the affected animal through induced discomfort or soreness. Moreover, there is no single effective treatment for this tumor, and it has a high recurrence probability [4,5,6,7]. It has been shown that the presence of the sarcoid is associated with the presence of genetic material of bovine papillomavirus types 1 and 2 and, less frequently, 13 (*BPV-1*, *-2* or *-13*) [5,8,9]. This virus belongs to a species-specific family of viruses attacking skin cells, *Papillomaviridae*, and the sarcoid is the only documented case of infection of an organism other than its default host [10,11]. The genome of *BPV* consists of double-stranded DNA in which the late genes (*L1* and *L2*) and early genes (*E1*–*E7*) can be specified. Late genes are responsible for the production of capsid proteins, while early genes are related to replication, transcription control and encode individual viral proteins, including transforming proteins [12].

The exact mechanism responsible for the formation of the sarcoid is not fully understood. It has been shown that the mere presence of viral genetic material in skin cells is not sufficient to generate a sarcoid [9,13]. However, it has been observed that sarcoids are most often formed in places where the skin has been previously traumatized [8]. On this basis, it has been hypothesized that the sarcoid forms as a consequence of an incorrect wound healing process, which may result from disturbances in the proper ECM composition of the skin tissue due to the presence of viral DNA [14]. Therefore, in the present study, we decided to analyze selected genes related to the ECM rearrangements and affecting the process of cell adhesion, which is dependent on the alterations of ECM properties. The current investigation also broadens mechanistic insights into the molecular basis of the lack of metastatic capacity pinpointed for this neoplasia. To the best of our knowledge, thoroughly elucidating the genetic background of multifaceted etiopathogenesis of epidermal and dermal sarcoid-related neoplasia in both equine in vivo and ex vivo models has provided, for the first time, strong empirical evidence for profound alterations in the molecular phenotypes determining intracellular pathways of ECM remodeling and cell adhesion. This might be tremendously helpful for future studies that aim to extensively exploring the epigenetic mechanisms underlying either the suppression/repression or restoration/recapitulation of molecular traits positively correlated with the sarcoid-dependent tumorigenic transformation of skin-derived cells in domestic horses. Such a collection of further studies might be especially suitable for assessing the capabilities of nuclear genomes inherited from neoplastic skin cell derivatives that can be epigenetically reprogrammed in equine somatic cell-cloned embryos and progeny. In turn, research focused on somatic cell nuclear transfer (SCNT)-based cloning might contribute to the development and optimization of the preclinical and clinical modalities of oncological treatments in domestic horses, as well as other equids afflicted with sarcoid-mediated cancerogenesis diagnosed within cutaneous and subcutaneous tissue compartments. For all the above-mentioned reasons, the present investigation sought to comprehensively compare the differences in gene expression patterns and their resultant impacts on the changes in ECM structure, not only between healthy skin tissue bioptates and the sarcoid tissue samples, but also between non-transfected dermal fibroblast cell lines and dermal fibroblast cell lines transfected with the gene encoding the BPV1-E1^E4 protein.

## 2. Results

### 2.1. Identified DEGs Belonged to ECM Remodeling and Cell Adhesion Pathways

**Figure 1:**
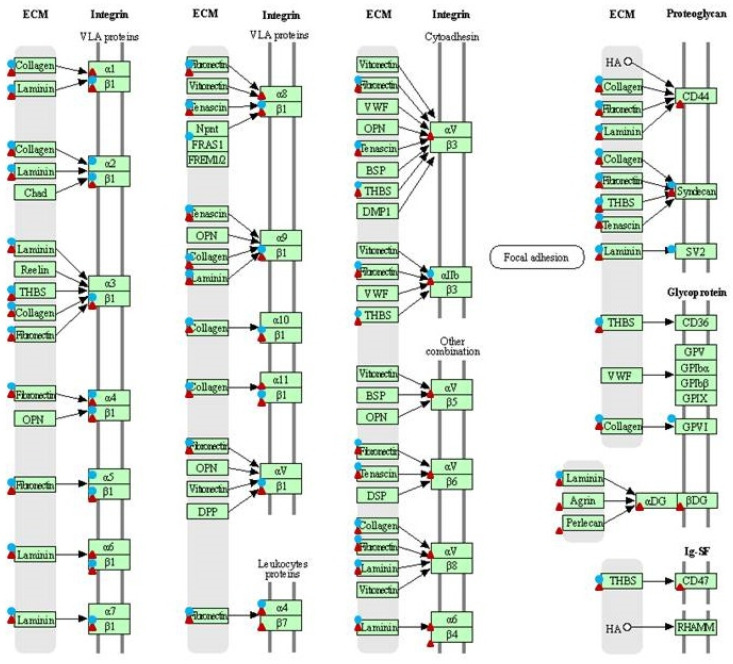
Differentially expressed genes (DEGs) related to ECM remodeling pathway (ecb04512). Circles—DEGs identified with the microarray analysis. Triangles—DEGs identified with the RNA-seq analysis (KEGG pathway database [15]).

**Figure 2:**
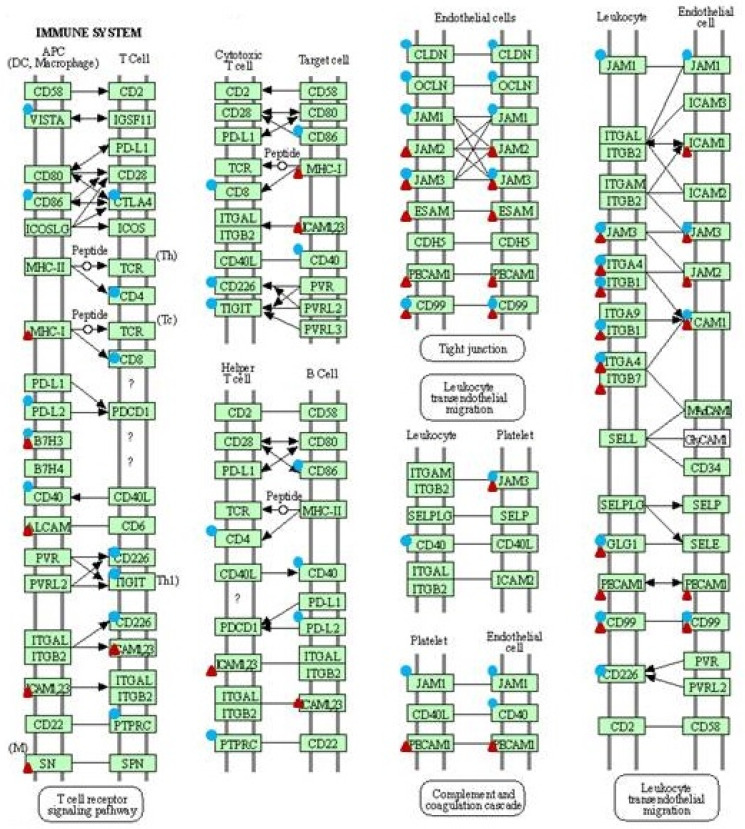
Differentially expressed genes (DEGs) related to cell adhesion pathway (ecb04514). Circles—DEGs identified with the microarray analysis. Triangles—DEGs identified with the RNA-seq analysis (KEGG pathway database [15]).

**Figure 3:**
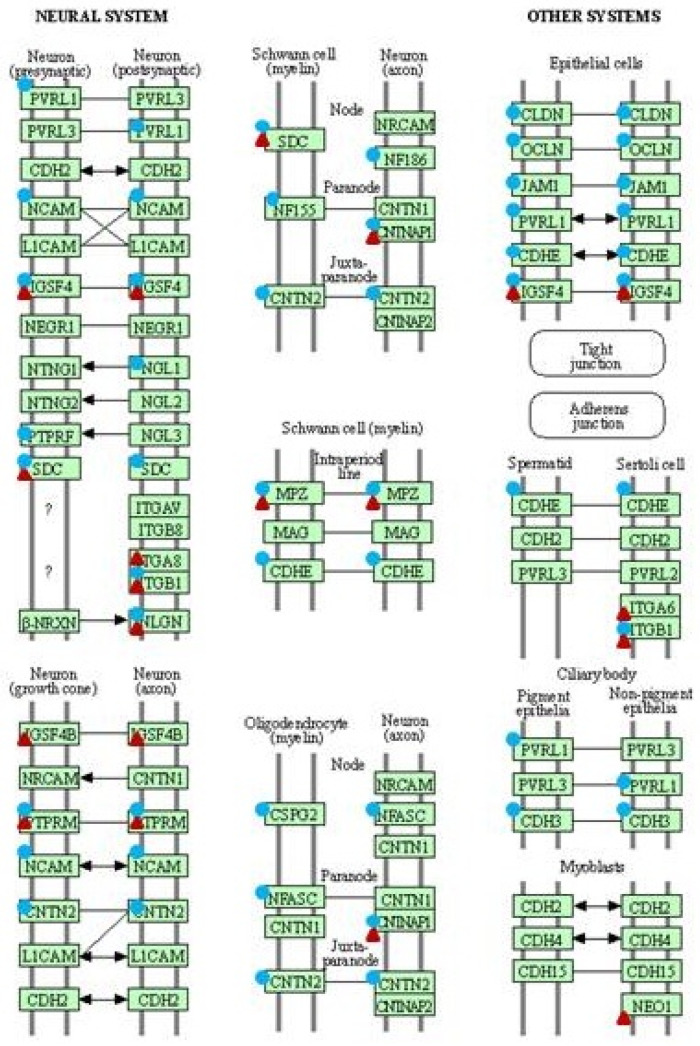
Differentially expressed genes (DEGs) related to cell adhesion pathway (ecb04514). Circles—DEGs identified with the microarray analysis. Triangles—DEGs identified with the RNA-seq analysis (KEGG pathway database [15]).

The pathway enrichment analysis of set of differentially expressed genes (DEGs), which occurred between dermal fibroblast cell lines transfected with gene construct coding for the BPV1-E1^E4 protein and control (i.e., non-transfected) fibroblast cell lines, allowed for the identification of 30 DEGs (*p*-value < 0.05) that belong to ECM remodeling pathway (as indicated by the false discovery rate; FDR < 0.000012) and 27 that belong to cell adhesion pathway (FDR < 0.03). The same analysis performed for the comparison of the sarcoid tissue samples and healthy skin showed a significant involvement of 29 DEGs in ECM remodeling (FDR < 0.001) and 44 DEGs in cell adhesion (FDR < 0.0001) pathways (Figure 1, Figure 2 and Figure 3).

### 2.2. Selection of DEGs Potentially Involved in Sarcoids Occurrence

**Figure 4:**
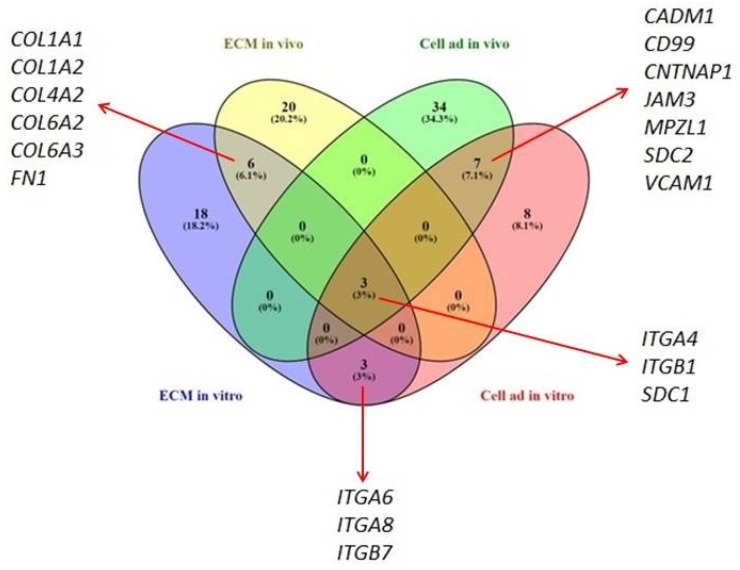
Venn diagram of common and unique differentially expressed genes (DEGs) following the comparisons of dermal fibroblast cell lines transfected with *BPV1-E1^E4* gene constructs and sarcoid tissue vs. control groups for ECM remodeling pathway (ECM in vitro and ECM in vivo, respectively); dermal fibroblast cell lines transfected with *BPV1-E1^E4* gene constructs and sarcoid tissue vs. their control groups for cell adhesion pathway (Cell ad in vitro and Cell ad in vivo, respectively) (Venny 2.1 BioinfoGP [16]).

The most numerous DEGs detected within both pathways were genes coding collagens, integrins, laminins and claudins (Table 1). In order to identify deregulated genes common for both in vivo and in vitro comparisons, a Venn diagram was used (Figure 4). Four panels of genes were compared, and we observed DEGs that were unique to each analysis and common gene set, modified regardless of in vitro or in vivo approaches. Seven DEGs involved in the cell adhesion pathway (*CADM1*, *CD99*, *CNTNAP1*, *JAM3*, *MPZL1*, *SDC2*, *VCAM1*) were detected as significant, regardless of the analyzed model.

Similarly, six DEGs belonging to the ECM matrix remodeling pathway (*COL1A1*, *COL1A2*, *COL4A2*, *COL6A2*, *COL6A3*, *FN1*) were frequently identified in sarcoid tissue explants as compared to healthy skin samples, groups of dermal fibroblast cell lines transfected with *BPV1-E1^E4* gene constructs, and control (i.e., non-transfected) dermal fibroblast cell lines (Figure 4). Moreover, *ITGA6*, *ITGA8* and *ITGB7* genes were detected as significantly differentially expressed and belonged to the ECM matrix and cell adhesion pathways. Interestingly, three genes (*ITGA4, ITGB1* and *SDC1*), whose expressions were significantly modified in both pathways, were detected in both in vivo and in vitro models of sarcoid-related tumorigenesis.

Based on the aforementioned findings, nine DEGs (*CADM1*, *CD99*, *CNTNAP1*, *FN1*, *JAM3*, *MPZL1*, *SDC1*, *SDC2*, *VCAM1*) were selected for a further analysis using real-time PCR.

### 2.3. Expression Patterns of Selected DEGs Evaluated Using qPCR

#### 2.3.1. The Genes Up-Regulated in Sarcoids and *BPV1-E1^E4* Transgenic Dermal Fibroblast Cell Lines

**Figure 5:**
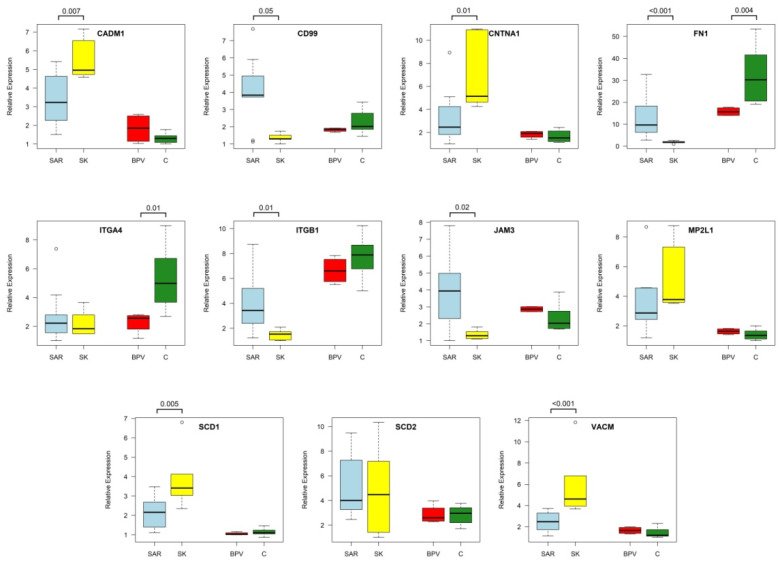
The differences in DEGs expression levels between analyzed groups of equine sarcoids (SAR), skin (SK) samples, control dermal fibroblast cell lines nucleofected with empty vectors (C), and dermal fibroblast cell lines nucleofected with *BPV-E4^E1* transgene (BPV) (R software v4.1 [17]).

The qPCR analysis confirmed a significant up-regulation of several genes in the sarcoid samples compared to healthy skin tissue. An increased expression level was observed for *CD99* (*p*-value < 0.0495); *FN1* (*p*-value < 0.0002); *ITGB1* (*p*-value < 0.0109); and *JAM3* (*p*-value < 0.0224). The greatest differences between the analyzed groups were detected for *FN1*, *CD99* and *JAM3* genes, as indicated by fold change (FC) at the levels of 7.43, 3.05 and 3.06, respectively (Figure 5).

#### 2.3.2. The Genes Down-Regulated in Sarcoids and *BPV1-E1^E4* Transgenic Dermal Fibroblast Cell Lines

Four genes—*CADM1*; *CNTNAP1*; *SCD1*; and *VCAM1*—were significantly down-regulated in sarcoid tumors (Figure 5). The lowest transcript level in sarcoids compared to healthy tissue was identified for *VCAM1* (*p*-value < 0.0010; FC −2.41) and *CNTNA1* (*p*-value < 0.0109; FC −2.10). For the two other genes, FC values were as follows: −1.83 for *SDC1* and −1.64 for *CADM1*.

For the *FN1* gene, a significant down-regulation of the expression level was detected in *BPV1-E1^E4* transgenic cell lines compared to control cell lines (*p*-value < 0.0040). The obtained difference was a −2.04-fold change. Similarly, the expression level in control cell lines was significantly higher for the *ITGA4* gene as compared to *BPV1-E1^E4* transgenic cells (*p*-value < 0.0161).

### 2.4. The Functional Enrichment Analysis of the Obtained Network

**Figure 6:**
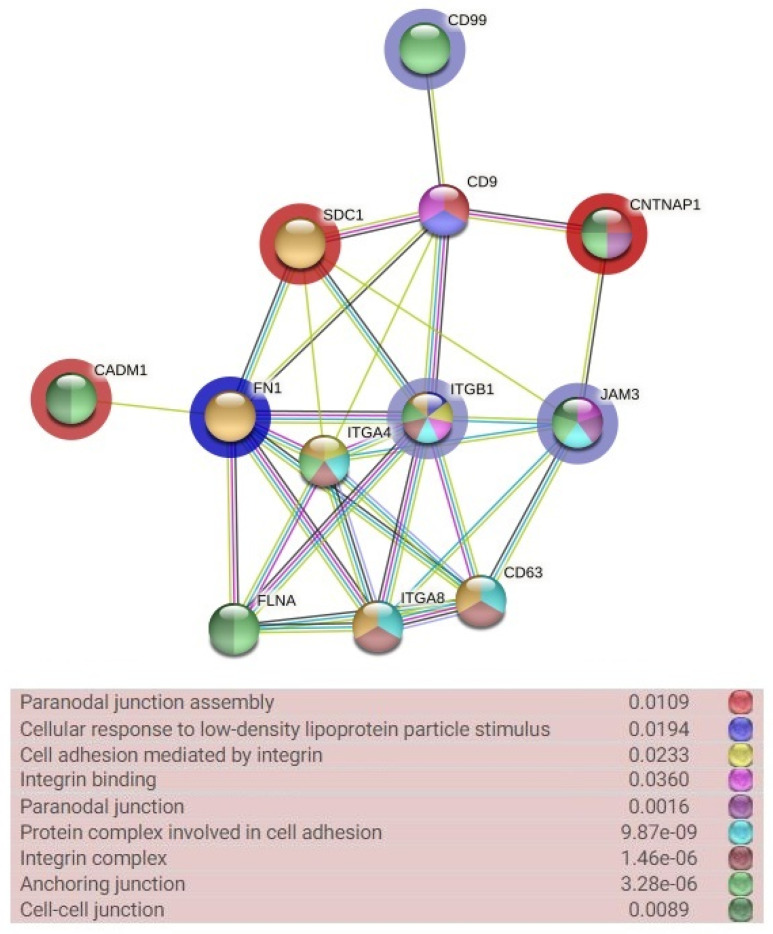
The gene ontology (GO) terms and interactions between set of chosen differentially expressed genes (DEGs) and their closest connected genes (String software [18] *Equus caballus* reference). The GO terms are marked in color, as shown in the figure legend (GOs are presented with their corresponding false discovery rates; FDRs). The blue and red areolas show fold change (blue—up-regulation; red—down-regulation).

The gene ontology (GO) analysis of genes that showed differential expressions confirmed their involvement in the anchoring junction (FDR < 0.0001), integrin complex (FDR < 0.0001) and protein complex involved in cell adhesion (FDR < 0.0001), as well as the paranodal junction (FDR < 0.0016), cell–cell junction (FDR < 0.0089) and integrin binding (FDR < 0.0360) (Figure 6). Among the genes involved in the most numerous GO terms were those identified as up-regulated (*JAM3* and *ITGB1*) and those identified as down-regulated (*CNTNAP1*), while *FN1* and *ITGB1* exhibited the highest number of interactions between genes. The analysis of the closest connections with other genes involved in the processes and not included in our analyzes indicated that *CD63*, *CD9*, *ITGA8* and *FLNA* genes can be candidate genes related to the ECM remodeling and cell adhesion during sarcoid growth and development. The *ITGA4* gene was also identified as strongly related to the majority of GO terms, but its differential expression was confirmed only in the in vitro model.

## 3. Discussion

ECM and cell adhesion molecules remodeling are considered as essential factors that lead to the formation, growth, and development of cancer cells. Therefore, both molecular pathways/extracellular matrix remodeling and cell adhesion were the subjects of our interest in equine sarcoid occurrence. High-throughput NGS data allowed us to narrow the searching area of candidate genes associated with molecular remodeling in horse skin cells leading to sarcoid formation. Among all identified DEGs, selected genes were either involved in both investigated pathways or belonged to one pathway. Nonetheless, they were detected not only in sarcoid tissue bioptates, but also in the ex vivo-expanded dermal fibroblast cells transfected with *BPV1-E4^E1* gene construct.

The detected genes with the greatest changes in expression levels were *FN1* (Fibronectin 1); *CD99* (Cluster of differentiation 99) and *JAM3* (Junctional Adhesion Molecule 3), which were all significantly up-regulated in sarcoid tissue compared to healthy skin. Fibronectin is a multifunctional extracellular matrix (ECM) glycoprotein that plays a key role in tissue repair via involvement in early and the late wound-healing responses [19]. Fibronectin, as a part of the extracellular matrix, binds a broad spectrum of other ECM proteins, including collagens, laminins, fibrinogen and fibrillins, syndecans and tenascin [20]. Thus, fibronectin regulates the composition of the extracellular matrix as well as attaching to other ECM molecules [21]. Moreover, the fibronectin matrix separates selected growth factors and related proteins, e.g., BMP1, VEGF and LTBP in order to control the cell signaling [22]. On the one hand, due to such a broad spectrum of molecular dependencies, fibronectin plays a critical role in cell growth, adhesion, migration and differentiation. On the other hand, the disruptions of the structure or function of fibronectin can lead to remarkable changes in ECM organization and result in a number of disorders in organisms, including cancer [23]. The increased expression of *FN1* gene has been reported in many types of cancer, including gastric [24], breast [25], thyroid [26], renal [27] and ovarian [28] cancers. Furthermore, in many cases, the up-regulation of *FN1* indicates a poor prognosis for patients [24,26,28]. The elevated expression level of fibronectin 1 is strongly related to modifications of extracellular space, and it contributes and promotes the spread, migration, proliferation and differentiation of cells [29]. It has been confirmed that the over-expression of the *FN1* gene inhibited apoptosis processes and promoted cell migration by regulating the NF-κB pathway [30]. During tumorigenesis, cell migration is activated by the increased expression of *FN1,* which up-regulates both *MMP9* and *MMP2* genes [30]. In the present report, more than a 7-fold increase in *FN1* gene expression in the sarcoid tissue compared to healthy skin was observed. Such high differences can indicate that ECM remodeling occurs, as in both human cancers and equine sarcoids. Interestingly, the previous study performed on sarcoids confirmed the significant over-expression of both *MMP2* and *MMP9* genes in the tumor tissue and in cell lines transfected with *BPV-1* gene construct [31]. These findings strengthen the hypothesis that one of the main mechanisms responsible for sarcoid formation can be the *FN1*–matrix metalloproteinase axis. The increased transcript level of *FN1* and both *MMP9* and *MMP2* genes can be considered as biological markers of sarcoid formation.

The up-regulation of the expression level of *CD99* gene, which encodes cell surface glycoprotein belonging to ECM matrix and is responsible for cell–cell adhesion, was also observed in sarcoids. CD99 protein is responsible for cell migration, differentiation and apoptosis [32], but its exact function is not fully understood. In some cancers, the over-expression of *CD99* increases migration and invasion [33], while in most cases, *CD99* up-regulation enhances cell–cell adhesion and apoptosis inhibiting tumor cell migration and metastasis [34]. The second mechanism of *CD99* regulation may occur during sarcoid development, which may explain its non-metastasizing nature.

In turn, the JAM3 protein, which regulates cells adhesion and communication between cells and ECM [35], is up-regulated in variety of cancers [36,37]. The over-expression of *JAM3* promotes migration and suppresses apoptosis. The study performed on renal cell carcinoma showed that the *JAM3* gene is critical for its tumor migration ability via the regulation of genes coding for N-cadherin, integrin β1 (ITGB1) and MMP2 [36]. The presented study confirmed the sarcoid-specific increase in the expression of not only the *JAM3* gene, but also the *ITGB1* gene, whose expression is considered to be a poor outcome marker during cancer prognosis [38]. It is contrary to the nature of sarcoids, which is a non-metastasizing tumor. However, the expression patterns pinpointed for *ITGB1* and *JAM3* genes may suggest that, although these genes are over-expressed, their overall expression level may be not high enough to affect sarcoid tissue or their effect may be altered by co-expression patterns of another genes.

It is noteworthy that the *VCAM1* gene experienced the greatest down-regulation in sarcoid tissue as compared to the healthy skin control group. This gene encodes vascular cell adhesion molecule-1, whose expression is specific for epithelial cells, but under such conditions as high level inflammation or chronic diseases, its expression is also found on the surfaces of other cell types, including cancer cells [39,40]. The VCAM1 protein plays an important role in the recruitment of leukocytes and their migration to various tissues, which has numerous applications in chronic inflammation and cancerous tumorigenesis [39]. Various studies indicate a strong association of the *VCAM-1* gene with the tumor development process, where it plays a key role in angiogenesis and supports metastasis [39,40]. Its influence on metastasis has been observed in numerous neoplasms. An example is the positive correlation identified in the up-regulation of the *VCAM-1* gene with breast cancer metastases in lungs. In epithelium ovarian cancer patients, the high expression of this gene was associated with a low chance of survival. This situation is similar in the case of colorectal cancer, where the over-expression of the *VCAM-1* gene is associated with metastasis and progression of this cancer [40]. Moreover, the over-expression of this gene has been also observed in other malignant neoplasms, such as gastric cancer, melanoma and lung cancer [39]. These data strongly facilitate the justification of our findings, revealing that the low expression profiles identified for the *VCAM-1* gene in sarcoid tissue samples are responsible for the failure of this non-malignant (benign) neoplasm to metastasize. This can be explained by the lack of activation of transcription of this important biomarker of the carcinogenesis process.

The second-most down-regulated gene is the *CNTNAP1* gene encoding the contyactin-associated protein 1 (caspr-1). This protein is an important component of paranodal junctions, and its mutations are mainly associated with neuropathies [41]. However, there are reports that this gene is related to clear-cell renal carcinoma. In this tumor, the expression of the *CNTNAP1* gene was positively associated with cancer-associated fibroblasts [42]. The detection of the deregulated expression of this gene in sarcoid tissue samples, as compared to healthy skin-derived explants, may provide the empirical evidence and mechanistic insights that, despite its mainly neurological connections, the *CNTNAP1* gene may be a valuable source of information on the molecular basis of sarcoid formation. However, the lack of more detailed studies of this gene in the context of tumors do not allow such broad conclusions to be drawn.

The *SCD1* gene that encodes the enzymatic protein designated as stearoyl CoA desaturase 1 is proven to be associated with the lipid metabolism of the cell by biocatalyzing the synthesis of monosaturated fatty acids (MUFAs) from precursors that are saturated fatty acids (SFAs) [43]. This contributes to the synthesis of the basic components of biological membranes, and signaling molecules and provides a source of energy needed for the functioning of the cell [43]. Research also shows that the *SCD1* gene is related to the positive regulation of autophagy. This gene also plays an important role in the development of various cancers. The deregulated expression of the *SCD1* gene is associated with many human neoplasms, which indicates its important role in the process of carcinogenesis [44]. It has been shown that its overexpression is related to the proliferation of neoplastic cells and metastasis. Furthermore, the positive regulation of the autophagy process does not occur in all types of neoplasms [43]. For example, the transcriptional repression of the *SCD1* gene in human hepatocellular carcinoma (HCC) cells was brought about the activation of the apoptosis processes induced by autophagy [43], and this increased the expression of *SCD1* gene in these cells, leading to a worse prognosis for patients. The differentiation of influences on the process of autophagy is explained by the heterogeneous structure of the neoplastic tissue [43]. The involvement of this gene in lipid metabolism is largely associated with the process of carcinogenesis. The participation of *SCD1* gene in the synthesis of MUFAs suggests that it has a function supporting the proliferation of cancer cells by supplying them with the energy and building components that they need. Research that was conducted on human breast cancer and murine Lewis lung carcinoma confirmed that the silencing the transcriptional activity of *SCD1* gene was related to a reduction in tumor cell proliferation [45]. The inhibition of the MUFAs synthesis process and the resulting reduction in cell proliferation may explain why sarcoids do not exhibit metastasis.

The last gene observed to have a decreased expression in sarcoid tissues relative to the control was *CADM1*. The low expression of this gene was detected in many neoplasms, with the exception of hematological tumors, in which the overexpression of this gene was observed [46,47]. It is assumed that the reduction in the expression of this gene takes place through the methylation of its promoter [46]. On this basis, it can be concluded that a similar mechanism occurs in skin cells infected with *BPV*. It was shown that the low level of expression of this gene is associated with the development of neoplasms, and can function as an indicator of poor prognosis in patients suffering from numerous neoplasms, such as those of the respiratory system, hepatocellular cancer, glioblastoma and neuroblastoma [46,48]. Moreover, it is noteworthy that, as a result of expediting/intensifying the onset and progression of proapoptotic pathways, the overexpression of the *CADM1* gene was found to inhibit migration and metastasis in gastric cancer, colon, prostate, and ovarian cancers, as well as different skin cancers, such as malignant melanoma or cutaneous squamous cell carcinoma [46,47,48]. Therefore, it can be concluded that, in the process of sarcoid formation, which is characterized by a low metastasis, the level of deregulation of this gene is not high enough to lead to the migration of its cells into the body.

The last analyzed gene was integrin α4β1 (*ITGA4*), which was found to be down-regulated in dermal fibroblast cell lines oncogenically transformed via nucleofection with the *BPV1-E1^E4* gene construct. Its over-expression is correlated with increased metastasis in ovarian and colon cancers. Furthermore, the abundant expression of the *ITGA4* gene has been found in melanoma cells characterized by its high capabilities to metastasize. Additionally, the transcriptional suppression noticed for this gene can lead to the inhibition of metastasis [49]. Low quantitative profiles estimated in the expression of the *ITGA4* gene in *BPV1-E1^E4* transgenic dermal fibroblast cells support these data and could be one of the reasons for the lack of metastasis in sarcoid tissue.

To sum up, in the present study, we analyzed a panel of genes that were responsible for ECM remodeling and cell adhesion pathways. Our findings strongly support the hypothesis that ECM-related genes are correlated with sarcoid formation. The deregulated expression of selected genes was found in both equine sarcoid tissue bioptates and adult cutaneous fibroblast cell (ACFC) lines, neoplastically transformed by nucleofection with a gene construct encoding the BPV1-E1^E4 protein. These genes were up- and down-regulated and, in some cases (*CD99*, *ITGB1*, *JAM3* and *CADM1*), the pinpointed phenotypic background differed from the backgrounds noticed for similar expression patterns in other cancerous (malignant) or noncancerous (benign) neoplasms, as indicated according to the available literature data.

## 4. Materials and Methods

### 4.1. The Use of High-Throughput Data to Establish Genes Involved in ECM Remodeling and Cell Adhesion Pathways

To establish DEGs involved in ECM remodeling and cell adhesion pathways, two sets of data obtained from our previous investigations were used (GSE193906 and GSE83430) [31,50]. The raw data annotated as GSE193906 were generated via NGS sequencing of two groups encompassing ACFC lines transfected with the *BPV1-E1^E4* gene construct and control (i.e., non-transfected) ACFC lines. The raw reads were mapped to the reference genome (EquCab3; assembly 102 Ensembl) using STAR software v2.7.8. The inter-group comparative analysis of identified DEGs was accomplished with the aid of Deseq2 software v3.14. According to this statistical software, the levels of significant differences occurring between experimental groups were adjusted to *p*-values < 0.05 after multiple testing corrections.

In order to obtain full insights into the transcriptomic modifications that can take place during sarcoid formation, a second set of data was used: GSE83430 [50]. The data denoted as significantly different (*p*-value < 0.05) were analyzed in a way similar to the previous work [50]; however, without a final filtering of genes by fold change (FC). Specifically, quality control was performed by normalizing the signal strength distribution followed by a correlation analysis and principal component analysis with the aid of the GeneSpring GX software, version 14.9 (Agilent Technologies, Santa Clara, CA, USA). The presence of inter-group significant diversity in the expression of genes between each pair of sarcoid bioptates and control skin tissue explants was confirmed by both Student’s *t*-test and FC estimation, which enables DEGs to be identified. Subsequently, the Benjamini–Hochberg procedure was used to calculate the adjusted *p*-values (false discovery rates; FDRs). The criteria of statistical significance were *p*-value < 0.05 and FC > 1.

The occurrence of significant variability between identified DEGs (at the levels of *p*-value < 0.05), which was proven for both comparisons (sarcoid bioptates vs. healthy skin explants and ACFC lines transfected with gene construct coding for BPV1-E1^E4 protein vs. control ACFC lines), was also thoroughly evaluated depending on the commitment of DEGs to and their over-representation in either ECM remodeling or cell adhesion pathways. This statistical evaluation of inter-group variability (at the levels of FDR < 0.05) was achieved by using David software (version 6.8) [51] based on the *Equus caballus* reference and KEGG database (Fisher’s exact test with Benjamini correction) [15]. String software v11.5 [18] with the *Equus caballus* reference was applied to identify protein interactions.

### 4.2. Collection of Tissue Samples

Skin tissue samples were collected post mortem from horses (near eye region) in a slaughter facility (n = 8). Tissue bioptates were collected in tubes filled with either RNAlater solution (Ambion; Thermo Scientific, Waltham, MA, USA) (for the purposes of RNA isolation) or cell culture medium comprised of Dulbecco’s Modified Eagle’s Medium (DMEM; Gibco, Thermo Scientific, Waltham, MA, USA) supplemented with 10% fetal bovine serum (FBS; Gibco, Thermo Scientific, Waltham, MA, USA) (for the purposes of establishing the primary cultures and resultant ACFC lines). Sarcoid tissues (n = 10) were collected during standard veterinary removal procedures. All procedures were approved by Polish law (The Polish Act on the Protection of Animals Used for Scientific or Educational Purposes of 15 January 2015, which implements Directive 2010/63/EU of the European Parliament on the protection of animals used for scientific purposes), and further approval by the Animal Ethics Committee was not mandatory.

### 4.3. Establishment of Primary Cultures and Nucleofection of Equine ACFCs

The procedures encompassing the ex vivo establishment and nucleofection of ACFC lines were comprehensively described in the study by Podstawski et al. [52]. Briefly, the primary cultures of horse skin-derived fibroblast cells followed by mitotically stable ACFC lines were established in compliance with the method developed and optimized by Tomasek et al. [53]. According to this method, the small pieces of dermal tissue explants were placed at the bottom of the culture dish filled with DMEM (Gibco, Thermo Scientific, Waltham, MA, USA) enriched with 10% FBS (Gibco, Thermo Scientific, Waltham, MA, USA) and incubated until the fibroblast cells outgrew the skin bioptates, started to spontaneously and vigorously migrate, and formed cell colonies at the bottom of the culture dish. Subsequently, the fibroblast cell lines were cultured under the conditions of 37 °C, 5% CO_2_ and 100% humidity until they reached 90% confluence followed by several passages leading to cell population doublings.

As thoroughly specified in our previous investigation [52], immediately after ACFC lines had reached 90% confluence, they were trypsinized, centrifuged in Tissue Culture Medium 199 (TCM 199; Sigma-Aldrich, Merck Life Sciences, Poznań, Poland), supplemented with 5% FBS (Gibco, Thermo Scientific, Waltham, MA, USA) and then subjected to in vitro transgenization by nucleofection using the T-REx system (Invitrogen, Thermo Scientific, Waltham, MA, USA) and *BPV1-E1^E4* gene construct. The cell transfection was performed with the aid of the Amaxa Nucleofector™ II Device (Amaxa Biosystems, Lonza, Medianus, Kraków, Poland) and by using a dedicated reagent kit as follows: Amaxa™ Normal Human Dermal Fibroblast-Adult (NHDF-Adult) and Nucleofector™ Kit (Lonza, CELLLAB, Warsaw, Poland). Positive selection of transgenic (i.e., efficiently nucleofected) ACFC lines was achieved by 7-day verification of their resistance to a cocktail of antibiotics composed of 200 µg/mL zeocin (Invitrogen, Thermo Scientific, Waltham, MA, USA) and 6 µg/mL blasticidin S (Thermo Scientific, Waltham, MA, USA). The cell nucleofectants that survived the zeocin/blasticidin S-dependent selection were classified as transgenic and used for further procedures.

### 4.4. Gene Expression Measurements Using Real-Time PCR Approach

RNA was isolated (skin samples n = 8; sarcoids n = 10) with the PureLink™ RNA mini kit (Invitrogen, Thermo Scientific, Waltham, MA, USA) using an additional DNase treatment on the columns (PureLink™ DNase Set; Invitrogen, Thermo Scientific, Waltham, MA, USA). The quality and quantity of the obtained genetic material were validated by electrophoretic separation (2% agarose gel) and with the Nanodrop 2000 spectrophotometer (Thermo Scientific, Waltham, MA, USA). The RIN values were estimated using TapeStation 200 (Agilent Technologies, Santa Clara, CA, USA) and scores for RINs ranged from 8.5 to 9.5. Next, 300 ng of total RNA was used to synthesize cDNA using the High-Capacity RNA-to-cDNA™ Kit (Applied Biosystems, Thermo Fisher Scientific, Waltham, MA, USA). Then, a real-time PCR reaction was performed. Each reaction was carried out in triplicate. The reactions were performed on the QuantStudio7Flex platform (Applied Biosystems, Thermo Fisher Scientific, Waltham, MA, USA), and the Sensitive RT HS-PCR EvaGreen Mix kit (A&A Biotechnology, Gdynia, Poland) was used according to the manufacturer’s protocol. Two genes were used as endogenous controls: β-actin (*ACTB*) and ubiquitin B (*UBB*) [54]. The obtained results were calculated by the ΔΔCT method [55]. The real-time PCR primer sequences that were used are presented in Supplementary Table S1.

## 5. Conclusions and Future Goals

For further investigations, a comprehensive deciphering of the molecular scenarios that are responsible for the onset and progression of ECM remodeling and cell adhesion, in both in vivo and in vitro research models of sarcoid-dependent tumorigenic transformation, might be a useful tool. These investigations might create the biological foundations to identify a desirable source of highly reprogrammable and dedifferentiable neoplastic derivatives of dermal tissue cells. These skin-derived sarcoid cells might provide donor cell nuclei, which display a strong capability to epigenomically reprogram their transcriptomic signatures in equine embryos generated by somatic cell cloning. The production of such cloned horse embryos, which are able to develop into conceptuses and progeny, might be a powerful strategy for designing in vivo and ex vivo biomedical models. These models can be used for the preclinical and clinical exploration of genetic and epigenetic mechanisms, which underly the processes of either remission or resumption of procancerous tumorigenesis of cutaneous and subcutaneous tissue compartments into sarcoids.

## Figures and Tables

**Table 1:** Identified differentially expressed genes (DEGs) related to ECM remodeling and cell adhesion pathways.

	ECM Remodeling		Cell Adhesion	
	cell lines transfected with *BPV1-E1^E4* gene and control lines	sarcoid tissue and healthy skin	cell lines transfected with *BPV1-E1^E4* gene and control lines	sarcoid tissue and healthy skin
Collagens	*COL11A1; COL1A1; COL1A2; COL4A1; COL5A1; COL5A2; COL5A3; COL6A2; COL6A3; COL6A6*	*COL1A1; COL1A2; COL2A1; COL4A1; COL4A2; COL6A1; COL6A2; COL6A3; COL9A1; COL9A2; COL9A3*	-	-
Integrins	*ITGA1; ITGA11; ITGA4;* *ITGA6; ITGA8; ITGB1; ITGB7*	*ITGA2; ITGA2B; ITGA4; ITGA5; ITGB1*	*ITGA4; ITGA6; ITGA8; ITGB1; ITGB7*	*ITGA4; ITGB1*
Laminins	*LAMA3; LAMA4; LAMA5; LAMC3*	*LAMA2; LAMB1; LAMB3; LAMB4; LAMC1*	-	-
Claudins	-	-	*-*	*CLDN14; CLDN16; CLDN17; CLDN2; CLDN34; CLDN4; CLDN9*

## Data Availability

The study used RNA-seq and cDNA microarray data previously submitted to GEO database (GSE193906 and GSE83430 accession numbers).

## Supplementary Materials

The following supporting information can be downloaded at https://www.mdpi.com/article/10.3390/ijms23126506/s1.

## Abbreviations

ACFC	Adult cutaneous fibroblast cell
BMP	Bone morphogenetic protein
BPV	Bovine papillomavirus
CADM1	Cell adhesion molecule
CD99	Cluster of differentiation 99 surface antigen
CLDN	Claudin
CNTNAP	Contactin-associated protein
CoA	Coenzyme A
COL	Collagen
DEG	Differentially expressed gene
ECM	Extracellular matrix
FC	Fold change
FDR	False discovery rate
FLNA	Filamin A
FN1	Fibronectin
GO	Gene ontology
HCC	Human hepatocellular cancer
ITGA	Integrin subunit α
ITGB	Integrin subunit β
JAM	Junctional adhesion molecule
KEGG	Kyoto Encyclopedia of Genes and Genomes
LAMA	Laminin subunit α
LTBP	Latent transforming growth factor-β-binding protein
MMP	Matrix metalloproteinase
MPZL1	Myelin protein zero-like protein 1
MUFA	Monosaturated fatty acid
NF-κB	Nuclear factor κ-light-chain-enhancer of activated B cells
NGS	Next-generation sequencing
PCR	Polymerase chain reaction
qPCR	Quantitative polymerase chain reaction
SCNT	Somatic cell nuclear transfer
SDC	Syndecan
SFA	Saturated fatty acid
VCAM	Vascular cell adhesion molecule
VEGF	Vascular endothelial growth factor
