# Supplementary material for: Tracking the Molecular Scenarios for Tumorigenic Remodeling of Extracellular Matrix Based on Gene Expression Profiling in Equine Skin Neoplasia Models
Source: Int J Mol Sci. 2022 Jun 10;23(12):6506. doi: 10.3390/ijms23126506 (PMC9223705; doi:10.3390/ijms23126506)
Supplement: Supplementary file 1 [file ijms-23-06506-s001.zip › ijms-1763503-supplementary.pdf]

Table S1. The differentially expressed genes analyzed using real-time PCR.

Gene	Accesion Number	Primers	Product Length [bp]
CADM1	ENSECAG00000015144	F: GGTAACTTGGGTGAGAGTGGA	145
		R: ATCCGAATGAGCTTTCCCCA	
CD99	ENSECAG00000039059	F: GCCATCTCTAGCTTTATTGCCT	100
		R: CGTTGGTGCTGTGATGGTTT	
CNTNAP1	ENSECAG00000024128	F: GTCCTCAACGACCAGCACT	195
		R: GCGGAAGTTATGGCGATAGG	
FN1	ENSECAG00000000701	F: ATCATCCTGAGCACACTGGT	200
		R: GGCAATAACTTCCAGGTCCC	
ITGA4	ENSECAG00000010500	F: GTCACGCTTTCCAGACAGC	135
		R: CTCGTAAATCAGAGGGCATTCC	
ITGB1	ENSECAG00000022498	F: GCCTACCTCTGCACGATGT	166
		R: CTGGGTGATGTCTTCTGGCT	
JAM3	ENSECAG00000015550	F: GAAGCCAGTACCTCCTGTGT	158
		R: ATCTGGGATTGGCTCTGGAG	
MPZL1	ENSECAG00000006964	F: ATCTTCGTGGCAAATGGGAC	118
		R: CAGAGGTGTCGGTCCCCT	
SDC1	ENSECAG00000014709	F: CTCTACCTCCATCGTGCCAG	232
		R: AGAGGTCTTGTGGTGGTCAG	
SDC2	ENSECAG00000008484	F: AGCTGACAACATCTCGACCA	171
		R: CTGGGTCCACGTTCCTTTCT	
VCAM1	ENSECAG00000016735	F: TCACTCAAAATGACACGGGG	173
		R: GGAGTCCGATTTCCTGGGA	
ACTB [54]	AF035774	F CCAGCACGATGAAGATCAAG	88
		RGTGGACAATGAGGCCAGAAT	
UBB [54]	AF506969	F GCAAGACCATCACCCTGGA	206
		R CTAACAGCCACCCCTGAGAC	

[54] Bogaert, L.; Van Poucke, M.; De Baere, C.; Peelman, L.; Gasthuys, F.; Martens, A. Selection of a Set of Reliable Reference Genes for Quantitative Real-Time PCR in Normal Equine Skin and in Equine Sarcoids. BMC Biotechnol. 2006, 6, 24. https://doi.org/10.1186/1472-6750-6-24.
